# Remarkable Case of Adipocere

**Published:** 1860-01

**Authors:** 


### ARTICLE XX.

### *Remarkable Case of Adipocere.*

AT a meeting of the New York Pathological Society, held Sept. 14th, Dr. Dalton presented a body which had undergone complete transformation into adipocere. As far as could be ascertained, the body was buried in 1832. It was found in a cemetery, or rather in a pit, in the upper part of the city, which was dug out for the reception of cholera patients. The bodies were placed in separate coffins, but not in separate graves. The coffin containing this body was found about twenty feet beneath the surface; underneath it were three tiers of coffins, and above it nine or ten. The uppermost tier of coffins was covered by three or four feet of solid earth. The soil directly under the coffin in which the body was found was very watery; above this level there was but little water, although the ground was very moist. The bones of the bodies contained in this pit, and in some cases the tendons, were melted together in a semi-fluid mass, the usual result of decomposition under ordinary circumstances.

At the water-mark there were several bodies converted into this adipocere. The specimen presented, however,

*Dental Cosmos (new series of Dental News-Letter,) August, 1859, p. 53.

was the most perfect. The hands and feet have been rattled off during transportation. When the body was first taken out, its color was almost precisely the same as now, (a dullish-white ;) if anything, it has become a little more brownish. It has now been exposed to the air for three months. Its consistency was decidedly less when first removed ; it was then like cheese of medium consistency, a mixture of the ductile and the brittle. In handling it great care had to be used. At that time it exhaled a tolerably strong odor, partly cheesy, ammoniacal, and earthy. Since that time the cheesy and the earthy odors have disappeared ; the ammoniacal smell, however, is still preceptible. In other respects it appears not to be altered in the least, and Dr. Dalton presumes it will remain in the same condition for years, for centuries, if properly taken care of.

The body is that of a large, fat woman, between 45 and 50 years of age, evidently a woman past the prime of life. The anterior parietes have sunk very much, particularly those of the abdomen, which appear to be in contact with the spinal column. The anterior portion of the chest is also collapsed. The change of animal tissue to the adipocere is absolutely complete in all the tissues, except the hair, nails and bones. The papillæ of the skin can be distinguished, but the other tissues cannot be made out.

The substance of which this mass is composed is known by the name of adipocere, or, as the French call it, "*graisse de cadavre*," (fat of dead bodies.) It is exceedingly light, so that one can easily raise the whole subject.

It is somewhat curious that all the bodies, which are reported as having undergone this degeneration, have been interred under precisely the same circumstances. The first case was observed in a similar pit at a cemetery in Paris.

The chemical composition of the substance is such that it is regarded as an ammoniacal soap, sometimes soap composed of ammonia and lime, in other instances almost exclusively a lime-soap. Orfila and Fourcroy, who had paid particular attention to this subject, assert that at first it is almost exclusively ammoniacal, the ammonia being supplied by the decomposition of the nitrogenized muscular tissue. This unites with the fat coming from the adipose tissue, which has become rancid, and produces an ammoniacal soap. Some French chemists regard it as a transformation of the muscles into oleic acid, so that adipocere may be produced by simple decomposition of the muscular tissue. The more generally received opinion is that it is simple decomposition of the muscular tissue into ammonia, which unites with the fat of the adipose tissue. This opinion is favored by the fact, that in almost every instance of this kind the bodies are those of extremely fat persons. Such was the fact in a case, the only case of the kind which Dr. Dalton has previously seen, where the body was that of an enormously fat man. Another reason which makes it probable that the fat must come from the adipose tissue is that, as Orfila ascertained, adipocere does not take place when the animal matter consists of muscular tissue only.

A body buried by *itself* will rarely be converted into adipocere, because the ammonia-compounds produced by the decomposition of the muscular substance are dissolved in the fluids of the body, and these fluids absorbed by the soil, and do not unite with the fat so as to form adipocere. But if a body is surrounded by other bodies, the bodies above, decomposing, produce ammoniacal fluids. These being washed down by the rain, filter through to the ninth or tenth coffin, the water of course in its descent becoming more and more loaded with ammonia, and this uniting with the fat of the lowermost bodies, produces adipocere. The bodies under the surface of the water do not undergo the transformation, probably because this substance is soluble in water.

This material, of which the body is composed, is very inflammable. A piece put on charcoal, placed before the flame of the blow-pipe, takes fire and is consumed readily, leaving scarcely any appreciable residue.

[*Phil. Med. and Surg. Reporter.*

